# Just-in-time Procedure Guides in Emergency Medicine

**DOI:** 10.5811/westjem.2022.2.53655

**Published:** 2022-05-10

**Authors:** Tracy Fennessy, Kendra Parekh, Ryan Walsh

**Affiliations:** Vanderbilt University Medical Center, Department of Emergency Medicine, Nashville, Tennessee

## BACKGROUND

The practice of emergency medicine (EM) requires that physicians be proficient in a vast array of procedures no matter how frequently or rarely they occur. The Accreditation Council for Graduate Medical Education and the American Board of Emergency Medicine have established a series of milestones regarding procedural performance in order to establish a minimum level of physician competency in the field of EM.^1^ Procedural skills inevitably degrade over time if not practiced regularly and, thus, maintenance of these skills requires continued practice in the clinical or simulated setting.^2^

A previously conducted needs assessment looked at current procedural skill practices by emergency physicians (EP) and found a significantly positive correlation between the frequency at which a skill was performed and the perceived confidence by physicians performing that particular skill. Additionally, they found that the vast majority of physicians would like to attend procedural training sessions if offered by their institution.^3^

In addition to a procedural skills curriculum, “just-in-time” (JIT) refresher training can be particularly useful for procedures that occur rarely in the emergency department (ED). A previous study indicated that JIT training led to improved trainee skills and confidence in performing procedures, from both the resident and supervisor perspective.^4^ A randomized controlled trial that evaluated transvenous pacemaker placement by EPs in a simulated setting found that a JIT intervention, which included both a refresher video and a step-by-step interactive checklist, significantly improved performance.^5^

## OBJECTIVES

To our knowledge, there is no currently published literature on the use of JIT procedural training guides in EM. Our first objective was to perform a needs assessment of EPs’ current JIT procedural resource usage. Our second objective was to examine the impact of creating a repository of easily accessible JIT procedural training guides with institution-specific information. Specifically, we sought to evaluate the effectiveness of the guides as a tool to increase knowledge and teach procedures, as well as the impact of the guides on physician confidence and procedural practice.

## CURRICULAR DESIGN

All EM faculty, fellows, and residents in the department were eligible to participate in this study.

### Survey Development

We used consensus decision-making to create a 12-question needs assessment focused on JIT resource utilization (Appendix A). Questions collected information regarding current resource use on shift to learn and teach procedures, barriers to using particular resources, frequency of specialty consultations for procedures due to lack of physician comfort in performing the procedure, and ideas for new resources. A five-point Likert scale was used to assess the degree to which physicians believed that their comfort level in performing procedures would change if they were provided with a detailed guide for a given procedure.

Five months after the implementation of the JIT procedure guides, we created a 14-question post-intervention survey (Appendix B) to assess the effectiveness of the guides as a tool to increase knowledge and teach procedures, as well as the impact of the guides on physician confidence and procedural practice. Five-point Likert scales were used to assess on average how often physicians required a JIT refresher prior to performing or teaching a given procedure, how often they used the JIT procedure guides, and how helpful they found these guides for both learning and teaching purposes. Physicians were also queried whether their procedural practice patterns had changed after the implementation of the guides. They were asked not only how their confidence had changed in performing procedures in the ED, but also whether their behavior had changed in terms of their decision to consult a specialist for a given procedure. We also assessed the quality of the procedure guides, most helpful features, and suggestions for improvement.

### Study Procedure

The study was granted an exemption by the medical center’s institutional review board. The online cross-sectional anonymous needs assessment survey was completed in December 2020 using REDCap (Vanderbilt University, Nashville, TN).^6^,^7^ The survey was disseminated using internal email distribution lists, and participants were given 10 weeks to complete the initial survey.

The needs assessment results directly informed the development of the JIT procedure guides and used Mayer’s principles of multimedia design.^8^ The 54 guides were distributed via the departmental website starting in December 2020 (Appendix C). Procedures covered were based on the Core Emergency Medicine Procedures as defined by the 2019 Model of the Clinical Practice of Emergency Medicine.^1^ The guides were created using PowerPoint (Microsoft Corporation, Redmond, WA), with a simple color-coded schema that was visually appealing and easy to follow. The content of the procedure guides included links to high-quality videos curated from the internet or author-created videos, detailed indications/contraindications, an overview of relevant anatomy, standard procedural steps along with (where applicable) steps specific to the procedural kits stocked in our ED, clear visual depictions of our ED kits, complications to anticipate, and aftercare recommendations (Appendix D). Announcements were made via email and at departmental meetings about the availability of the JIT procedure guides.

In May 2021 the anonymous, online post-intervention survey was distributed via internal email distribution lists. Participants were given three weeks to complete the post-survey.

### Data Analysis

We performed descriptive statistics and compared resident and faculty responses using Fisher’s exact testing due to the small sample size. All statistical analysis was performed using SPSS 27 (IBM Corp,, Armonk, NY)). Two authors performed thematic analysis of the responses to the two post-implementation survey questions, which asked about the most helpful features of the procedure guides and suggestions for improvement.

## IMPACT/EFFECTIVENESS

### Needs Assessment Results

**Figure 1:**
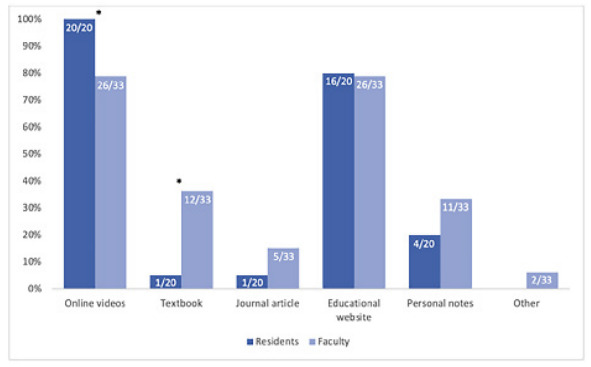
Percentage of residents and faculty indicating current just-in-time resource use on shift. * Indicates statistically significant difference between resident and faculty responses (P-value <0.05).

The overall response rate for the initial needs assessment survey was 49.5% (53/107): residents 51.3% (20/39) and faculty 48.5% (33/68). The majority of survey respondents reported using online videos (86.8%, 46/53) and educational websites (79.2%, 42/53), with far fewer using personal notes (28.3%, 15/53), textbooks (24.5%, 13/53), or journal articles (11.3%, 6/53) for JIT procedural guidance. There was a statistically significant difference between the percentage of residents and faculty who reported using videos (100% ([20/20] of residents vs 79% [26/33] of faculty, *P*<0.05) and textbooks (5% [1/20] of residents vs 36% [12/33] of faculty, *P*<0.05] as JIT resources on shift (Figure 1).

**Figure 2:**
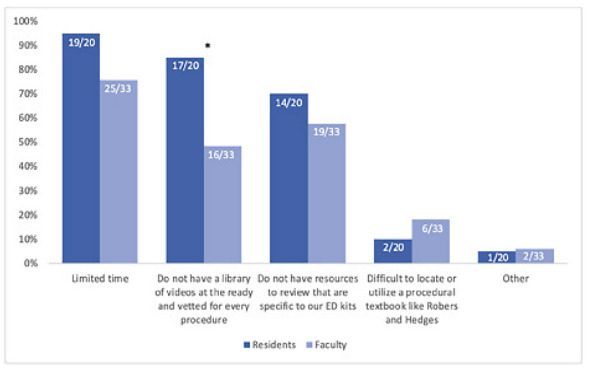
Percentage of residents and faculty indicating barriers to just-in-time resource utilization on shift. * Indicates statistically significant difference between resident and faculty responses (*P*-value <0.05). *ED*, emergency department.

The most common barriers to using JIT resources included limited time (83%, 44/53); resources not being specific to available procedural kits (62.3%, 33/53); and lack of curated, high-quality video resources (62.3%, 33/53). There was a statistically significant difference between the percentage of faculty and residents who identified a lack of a video library as a barrier (85% [17/20] of residents vs 48% [16/33] of faculty, *P*<0.01) (Figure 2). At least once within the last year, a majority of respondents (58.5%, 31/53) thought about performing a core EM procedure but ultimately did not do so due to lack of comfort in performing the procedure, and 88.7% (47/53) indicated their comfort level in performing a procedure would increase or significantly increase if detailed online procedure guides were available.

### Post-Implementation Survey Results

The overall response rate for the post-intervention survey was 29% (31/107): residents 31% (12/39), and faculty 28% (19/68). The majority (58.3%, 14/24) indicated that they sometimes needed a refresher during their clinical shift prior to performing a procedure. When they required a refresher, 25% (6/24) of faculty and residents always used the developed JIT procedure guides: 37.5% (9/24) often used them; 29.2% (7/24) sometimes used them; and only 8.3% (2/24) rarely or never used them. Most respondents used the guides to improve general procedural knowledge (83.3%, 20/24) and to teach procedures in the ED (75%, 18/24).

The vast majority of respondents (95.8%, 23/24) found the procedure guides to be very helpful in increasing their procedural knowledge, while one respondent (4.2%), found them to be somewhat helpful; (faculty 100% [15/15] very helpful; residents 88.9% [8/9] very helpful, and 11.1% [1/9] somewhat helpful). The majority of faculty also found the guides to be very helpful for teaching procedures (86.7%, 13/15), with the remainder neutral (13.3% (2/15). Rating the quality of the guides, faculty unanimously found them to be excellent (100%, 15/15), while the majority of residents (75%, 6/8) found them to be excellent, and some (25%, 2/8) found them to be very good. A minority (22.6%, 7/31) of survey respondents indicated that they had not yet used the procedure guides, and of those respondents 85.7% (6/7) had not yet needed a JIT refresher. Only one respondent (14.2%, 1/7) indicated they had difficulty accessing the guides on the website.

The Table displays all themes, theme frequency, and representative comments to the questions “What are the most helpful (or best) features of the procedure guides?” and “What suggestions do you have for improving the procedure guides?”

Since the implementation of the guides, the majority of residents (75%, 9/12) and faculty (68%, 13/19) felt their confidence in performing procedures had increased or significantly increased. Importantly, 16% (5/31) of respondents indicated they had changed their procedural practice pattern and performed a core EM procedure, covered by our JIT procedure guides, that they previously would have asked a consultant to perform.

## DISCUSSION

Our needs assessment indicated there is a clear need to have a readily accessible resource available for the purpose of JIT learning and teaching of EM procedures. While most physicians surveyed use online videos and educational websites, we found that a higher proportion of faculty use textbooks as a JIT resource relative to resident trainees (Figure 1). This could represent a broader pattern between different generations of learners and reinforces that educational interventions need to be targeted to the populations that are going to be using the resources provided.

We attempted to tackle the fact that different groups of learners not only have different learning needs when it comes to JIT procedural refreshers, but they also perceive different barriers. The procedure guides were an attempt to bridge this gap and meet the needs of multiple groups of learners by pulling useful aspects of each type of resource, including succinct text, clear visual depictions, and links to online videos.

One recurring theme that we found from respondents was that the guides were clear and concise. In the interest of keeping these guides concise, we chose to highlight only the primary procedural techniques and not make the guides all-inclusive. An additional theme from respondents was that it was helpful that we displayed and photographed, when applicable, the procedural kits specific to our ED. In some instances, we videotaped how to perform the procedure using our site-specific kits and included links to these videos in the guides. We believe that the ability to make these guides as specific to the institution as possible helped to increase their usefulness.

## LIMITATIONS

Limitations of the study include a low survey response rate and a short, five-month intervention period. This decision was made to ensure we maintained the same cohort of faculty, fellows, and residents prior to graduation. We felt, however, that the benefit of keeping the same cohort of physicians in the pre- and post-surveys justified the short implementation timeline. In addition, the project was conducted at a single academic institution, which may limit the generalizability of the results to other institutions. Further helpful areas of study would include data collection on procedure performance and procedural numbers to measure change in practice patterns of individual EPs, as well as consideration of patient outcomes data after the implementation of the JIT procedure guides.

## CONCLUSION

There is a clear need to have a readily accessible resource available for the purpose of just-in-time learning and teaching of EM procedures. Our results indicate that having clear, concise, readily accessible, and institution-specific JIT procedure guides can not only increase physicians’ confidence in their ability to perform core EM procedures in the ED, but also change physician behavior by potentially leading to reduced specialist consultation for these procedures, which may lead to a reduction in overall ED length of stay. Our guides represent an innovative resource that may appeal to multiple generations of emergency physicians, as they combine the most useful aspects of multiple resources including textbooks with more modern digital resources such as online videos.

## Supplementary Information









## Figures and Tables

**Table:** Thematic analysis of respondent comments on the best features of the procedure guides and suggestions for improvement.

Theme	Theme Frequency	Representative Comments
Best Features		

Clear format	42.3% (11/26)	“Very clear” “Very organized and straightforward”
Just-in-time access	26.9% (7/26)	“Are not hard to find” “Easily accessible”
High-yield details	23.1% (6/26)	“Focus on the high impact details” “The comprehensiveness of them”
Institution-specific	7.7% (2/26)	“Utilize supplies we are all familiar with in our own department”

Suggestions for Improvement		

Further improve ease of access	55.6% (5/9)	“Mobile app version” “The Emergency Medicine website could be optimized to make the guides more quickly accessible”
More guides	44.4% (4/9)	“Would love even more” “Focusing on more common procedures”
