# Supplementary material for: Just-in-time Procedure Guides in Emergency Medicine
Source: West J Emerg Med. 2022 May 10;23(3):353–7. doi: 10.5811/westjem.2022.2.53655 (PMC9183769; doi:10.5811/westjem.2022.2.53655)
Supplement: Supplementary file 1 [file wjem-23-353-s001.pdf]

Procedural Practice Survey

This is a needs assessment for procedural learning and teaching. All responses are anonymous.

Thank you for your time!

Select your current level of experience:

- ◯ PGY 1
- ◯ PGY 2
- ◯ PGY 3
- ◯ Less than 5 years since residency graduation
- ◯ 5-10 years since residency graduation
- ◯ More than 10 years since residency graduation

What tools do you currently use on shift prior to performing/teaching a procedure? Select all that apply.

- ☐ Online videos (ex. Youtube, Vimeo, etc.)
- ☐ Textbook (ex. Roberts and Hedges)
- ☐ Journal article
- ☐ Educational website
- ☐ Personal notes
- ☐ Other

If you answered "other" to the question above, please provide your response here:

____

While on shift, what barriers do you have to using resources to teach/learn procedures? Select all that apply.

- ☐ Limited time
- ☐ Don't have a library of videos at the ready and vetted for every procedure
- ☐ Don't have resources to review that are specific to our VUMC kits
- ☐ Difficult to locate or utilize a procedural textbook like Roberts and Hedges
- ☐ Other

If you answered "other" to the question above, please provide your answer here:

____

Within the last year, how many times have you thought about performing a procedure but did not do so due to discomfort with performing the procedure?

- ◯ 0
- ◯ 1-5
- ◯ 6-10
- ◯ 11-15
- ◯ >15

Which procedures were they?

____

For a given procedure, if you had a detailed procedure guide (similar to those now available on our website), how would your comfort level in performing the procedure change?

- ◯ Significantly decrease
- ◯ Decrease
- ◯ Stay the same
- ◯ Increase
- ◯ Significantly increase

Are there any procedures in particular you would want a procedure guide like this for?

____

How helpful would it be to have a training cart available in the service center with practice kits for ED procedures? (ex. pigtail chest tube kit, large volume paracentesis kit, etc.)

- ◯ Not at all helpful
- ◯ Slightly helpful
- ◯ Helpful
- ◯ Very helpful
- ◯ Extremely helpful

What methods of training do you currently use to maintain your own procedural comfort?	__________________________________
What method(s) would you find most helpful to maintain your procedural skills? Select all that apply.	☐ Faculty specific trainings<br>☐ Learning in conjunction with resident training on super Tuesdays<br>☐ Video recordings of resident procedural didactic sessions<br>☐ Podcast or procedural newsletter<br>☐ Other
If you answered "other" to the question above, please provide your answer here:	__________________________________
If in person procedural skills training for faculty is offered, how frequently would you like the training offered?	◯ Weekly<br>◯ Monthly<br>◯ Biannually<br>◯ Annually
Are there any other resources you would find helpful to have available when teaching learners procedures in the ED?	__________________________________
