# Supplementary material for: Just-in-time Procedure Guides in Emergency Medicine
Source: West J Emerg Med. 2022 May 10;23(3):353–7. doi: 10.5811/westjem.2022.2.53655 (PMC9183769; doi:10.5811/westjem.2022.2.53655)
Supplement: Supplementary file 2 [file wjem-23-353-s002.pdf]

Procedure Guides Post-Intervention Survey

This is a post-intervention survey to evaluate usage and helpfulness of the procedure guides published on our website. All responses are anonymous.

Thank you for your time!

Select your current level of experience:

- ◯ PGY1
- ◯ PGY2
- ◯ PGY3
- ◯ Less than 5 years since residency graduation
- ◯ 5-10 years since residency graduation
- ◯ More than 10 years since residency graduation

Since the procedure guides have been uploaded to the website, have you used them?

- ◯ Yes
- ◯ No

Over the past month, on average how often during your clinical shifts did you need a just in time refresher prior to performing/teaching a given procedure?

- ◯ Never
- ◯ Rarely
- ◯ Sometimes
- ◯ Often
- ◯ Always

Of the cases that you did need a just in time refresher prior to performing/teaching a procedure, on overage how often did you use the procedure guides on the website as opposed to a different resource?

- ◯ Never
- ◯ Rarely
- ◯ Sometimes
- ◯ Often
- ◯ Always

In which of the following ways have you used the procedure guides?
(select all that apply)

- ☐ To teach procedures in emergency department
- ☐ To teach in the classroom setting
- ☐ To teach in the simulation setting
- ☐ For reference to improve general procedural knowledge
- ☐ Other

If you answered "other" to the question above, please provide your answer here:

____

How helpful have you found the procedure guides to learn / increase your own knowledge for a particular procedure?

- ◯ Very unhelpful
- ◯ Somewhat unhelpful
- ◯ Neutral
- ◯ Somewhat helpful
- ◯ Very helpful

How helpful have you found the procedure guides to teach a particular procedure?

- ◯ Very unhelpful
- ◯ Somewhat unhelpful
- ◯ Neutral
- ◯ Somewhat helpful
- ◯ Very helpful

How would you rate the quality of the procedure guides?

- ◯ Poor
- ◯ Fair
- ◯ Good
- ◯ Very good
- ◯ Excellent

Since the implementation of the procedure guides, how has your confidence in performing procedures in the emergency department changed?	◯ Significantly decreased<br>◯ Decreased<br>◯ Unchanged<br>◯ Increased<br>◯ Significantly increased
Since the implementation of the procedure guides, have you performed a procedure in the emergency department that you previously would have asked a consultant to perform instead?	◯ Yes<br>◯ No
Which procedure(s)?	__________________________
What are the most helpful (or best) features of the procedure guides?	__________________________
What suggestions do you have for improving the procedure guides?	__________________________
Why have you not utilized the procedure guides yet? (select all that apply)	☐ Haven't had the need<br>☐ Don't know where to find them<br>☐ Difficult to access them on the website<br>☐ I have looked at them but don't like the procedure guides<br>☐ Other (please explain)
If you answered "other" to the question above, please provide your answer here:	__________________________
