# Supplementary material for: Just-in-time Procedure Guides in Emergency Medicine
Source: West J Emerg Med. 2022 May 10;23(3):353–7. doi: 10.5811/westjem.2022.2.53655 (PMC9183769; doi:10.5811/westjem.2022.2.53655)
Supplement: Supplementary file 3 [file wjem-23-353-s003.docx]

**Index List of JIT Procedure Guides**

1. A-line - Femoral
2. A-line - Radial
3. Arthrocentesis - Ankle
4. Arthrocentesis - Elbow
5. Arthrocentesis - Knee
6. Arthrocentesis - Wrist
7. Auricular Hematoma Repair
8. Blakemore-Minnesota Tube Placement - VUMC kit
9. Bleeding AV Fistula-Graft Repair
10. Breech Delivery
11. Compartment Pressure Measurement
12. Corneal Foreign Body Removal
13. Distal Radius Fracture - Reduction
14. Elbow Dislocation - Reduction
15. Felon Drainage
16. G-tube (Mini Button) Replacement
17. Hip Dislocation-Reduction
18. Intra-articular Lidocaine (IAL) - Shoulder
19. Knee Dislocation - Reduction
20. Lateral Canthotomy - Inferior Cantholysis
21. Nailbed Injury Repair
22. Nerve block - Head/Face - Ear
23. Nerve block - Head/Face - Supraorbital block
24. Nerve block - Head/Face - Infraorbital block
25. Nerve block - Head/Face - Inferior alveolar block
26. Nerve block - Head/Face - Mental nerve block
27. Nerve block - Head/Face - Occipital nerve block
28. Nerve block - Upper Extremity - Digital nerve block
29. Nerve block - Upper Extremity - Hematoma block
30. Nerve block - Upper Extremity - Median nerve block
31. Nerve block - Upper Extremity - Radial nerve block
32. Nerve block - Upper Extremity - Ulnar nerve block
33. Nerve block - Lower Extremity - Fascia iliaca block
34. Nerve block - Lower Extremity - Popliteal / distal sciatic nerve block
35. Nerve block - Lower Extremity - Posterior tibial nerve block
36. Nursemaid’s Elbow - Reduction
37. Paracentesis - Diagnostic
38. Paracentesis – Large Volume – VUMC kit
39. Paronychia Drainage
40. Pericardiocentesis
41. Peritonsillar Abscess Drainage
42. Pigtail Chest Tube – Standard
43. Pigtail Chest Tube – VUMC kit
44. Priapism management
45. Resuscitative Hysterotomy
46. Shoulder Dislocation - Reduction
47. Shoulder Dystocia
48. Splint Guide
49. Thoracentesis – VUMC kit
50. TMJ Dislocation - Reduction
51. Transcutaneous Pacing
52. Transvenous Pacing
53. Umbilical Vein Catheterization
54. Word Catheter Placement
