# Supplementary material for: Just-in-time Procedure Guides in Emergency Medicine
Source: West J Emerg Med. 2022 May 10;23(3):353–7. doi: 10.5811/westjem.2022.2.53655 (PMC9183769; doi:10.5811/westjem.2022.2.53655)
Supplement: Supplementary file 4 [file wjem-23-353-s004.pdf]

EMERGENCY MEDICINE PROCEDURES

PIGTAIL CHEST TUBE – VUMC KIT

Tracy Fennessy, MD, Ryan Walsh, MD

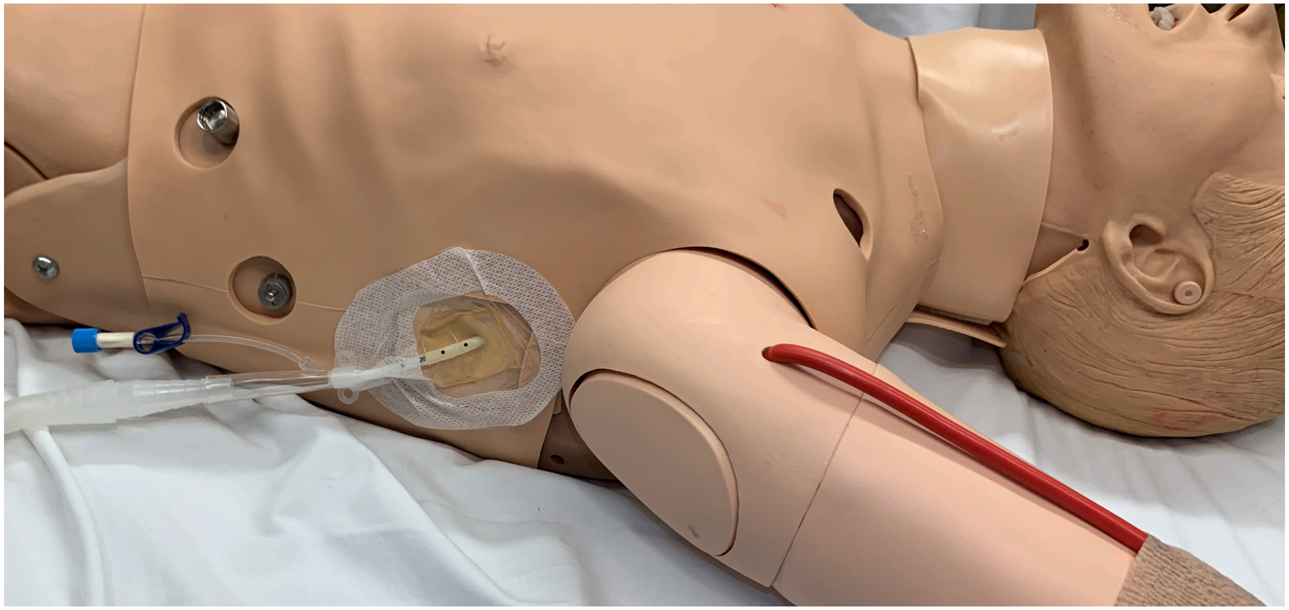

Video:
https://www.youtube.com/watch?v=7a988HEZful&feature=youtu.be&ab_channel=VUMCEmergencyMedicine
(Dr. Fennessy and Dr. Walsh – VUMC Kit)

PIGTAIL CHEST TUBE – VUMC KIT
INDICATIONS & CONTRAINDICATIONS

Indications (stable patient with one of the following):

1) Pneumothorax (spontaneous, traumatic, iatrogenic)
- Large (i.e. >3 cm between pleural line and chest wall at the apex)
- Failure of trial of oxygenation and observation
- Or recurrent episode

2) Hemothorax (relatively small not requiring large bore chest tube)

3) Pleural effusion

Absolute contraindications	Relative contraindications
1) Clinically unstable	1) Anticoagulation or coagulopathy
2) Tension physiology	2) Overlying infection
3) Massive hemothorax	3) Bleeding diathesis

Measuring pneumothorax size (American College of Chest Physicians)

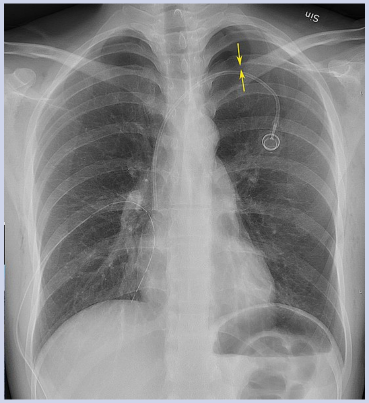

Apex to cupola distance:
- Small: < 3 cm
- Large: ≥ 3 cm

Note:

Clinically stable patients with a first episode of primary spontaneous pneumothorax (PSP) in whom the pneumothorax is small (<3 cm) should be treated with supplemental oxygen and observation and be discharged if appropriate.

PIGTAIL CHEST TUBE – VUMC KIT
ANATOMY

Landmarks / Boundaries (the "Zone of Safety")

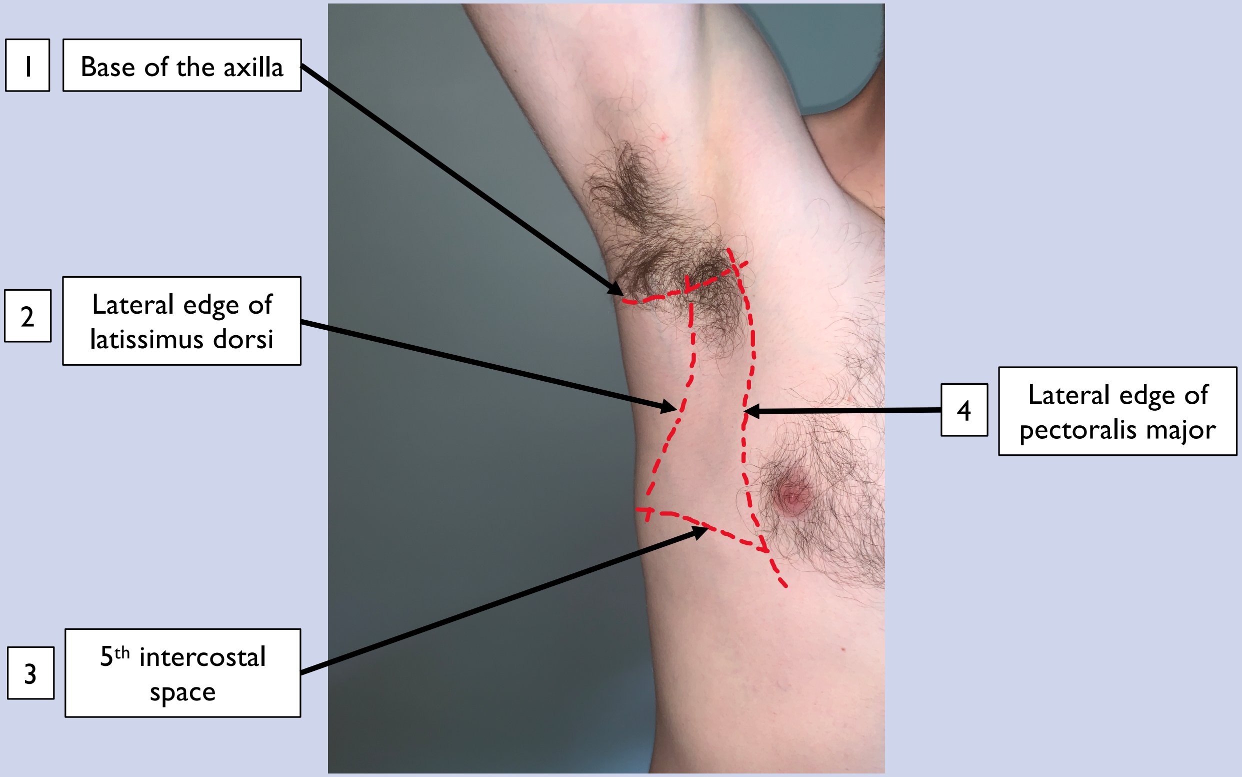

Neurovascular bundle anatomy

Ensure your angle of entry into the pleural space is ***__over the rib__***, to avoid the neurovascular bundle that runs under the rib

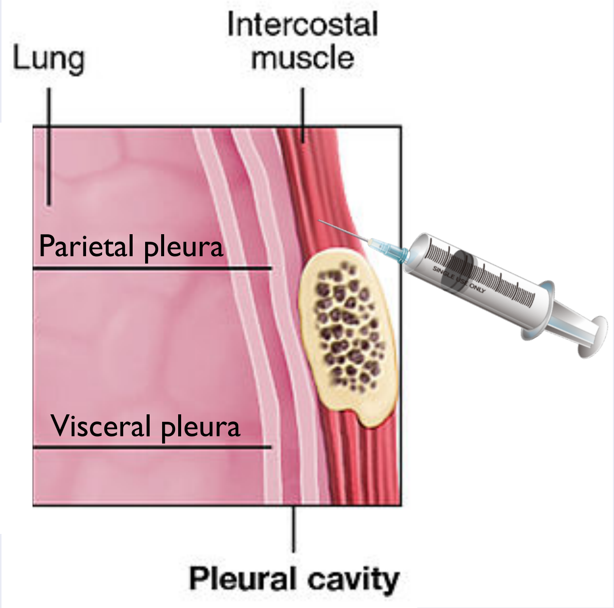

PIGTAIL CHEST TUBE – VUMC KIT
PREP

Supplies

1) Sterile gloves

2) Sterile gown

3) Facemask

4) Chest tube atrium (drainage box)

5) VUMC pigtail chest tube kit:

- Chloraprep
- Sterile drape
- Lidocaine (+syringe, draw up needle, infiltration needle)
- Finder needle (+syringe)
- Wire
- Scalpel
- Gauze
- Small dilator
- Large dilator
- Metal trocar introducer
- "Pigtail" chest tube
- Suture + needle driver
- Tegaderm dressing

1
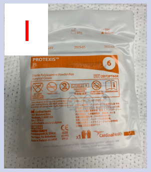

2
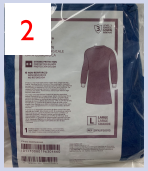

3
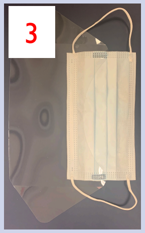

4
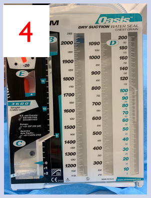

5
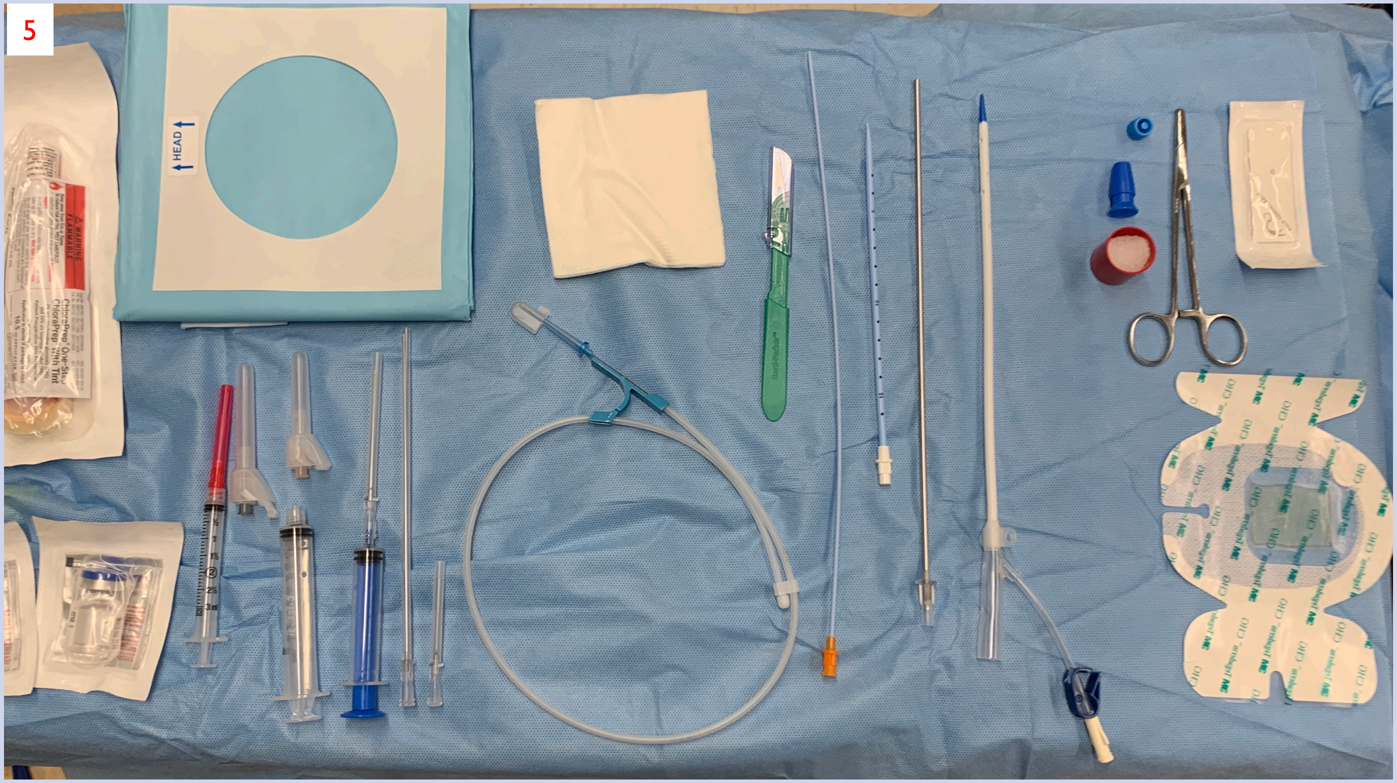

PIGTAIL CHEST TUBE – VUMC KIT
INITIAL SETUP

Cleaning and draping

1) Prep a large area with chloraprep

(all the way midline to sternum, up into axilla, and down to lower ribs)

2) Drop all supplies onto sterile field

3) Drape in a sterile fashion
+ put on sterile gloves

4) Anesthetize the site
- Local wheel, then…
- Inject 10 cc deeper along the trajectory of the chest tube insertion

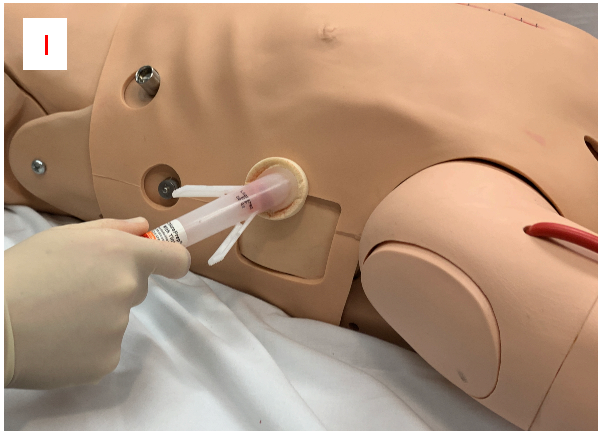

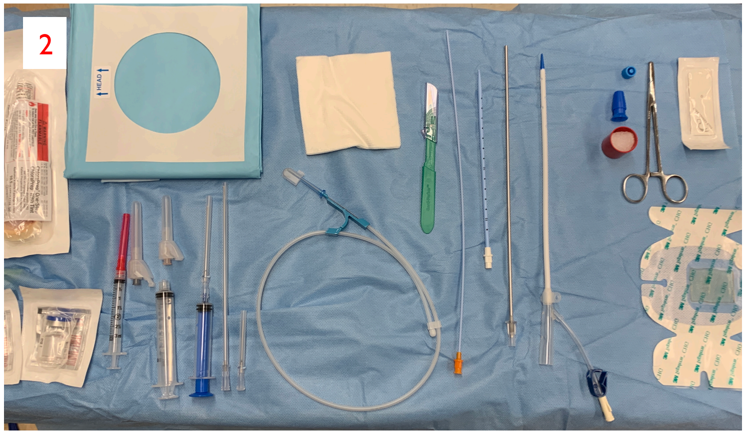

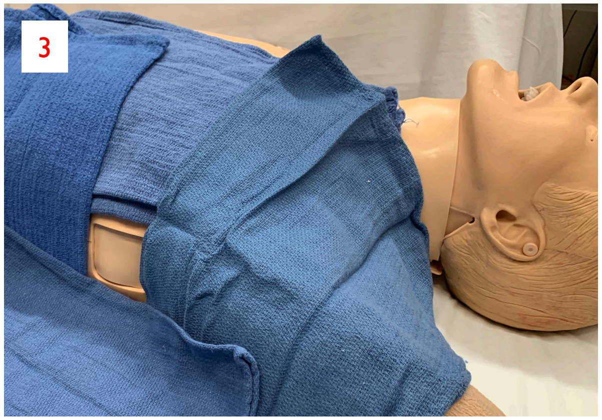

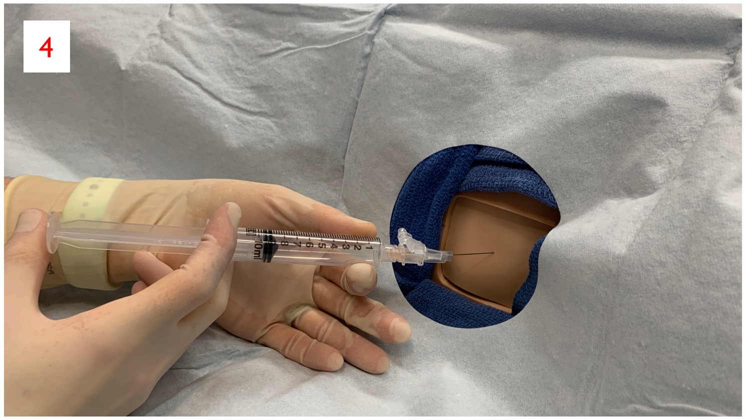

PIGTAIL CHEST TUBE – VUMC KIT
TECHNIQUE

Step 1: Insert the finder needle

Insert the finder needle over the rib, aspirating as you go

You know you're in the pleural space when either:

- If you start with an empty syringe → will get a "pop" of air
- If you start with sterile saline/lidocaine in the syringe → will see bubbles

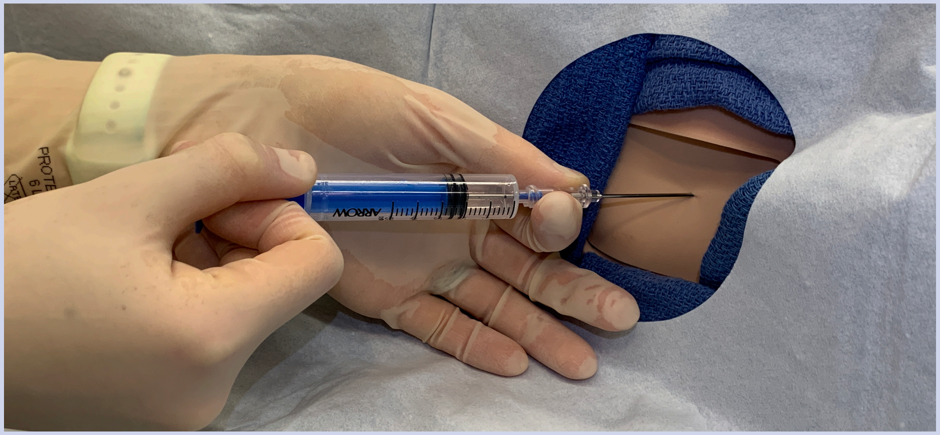

Step 2: Insert the wire

Anchor/hold the finder needle in place

Thread the wire

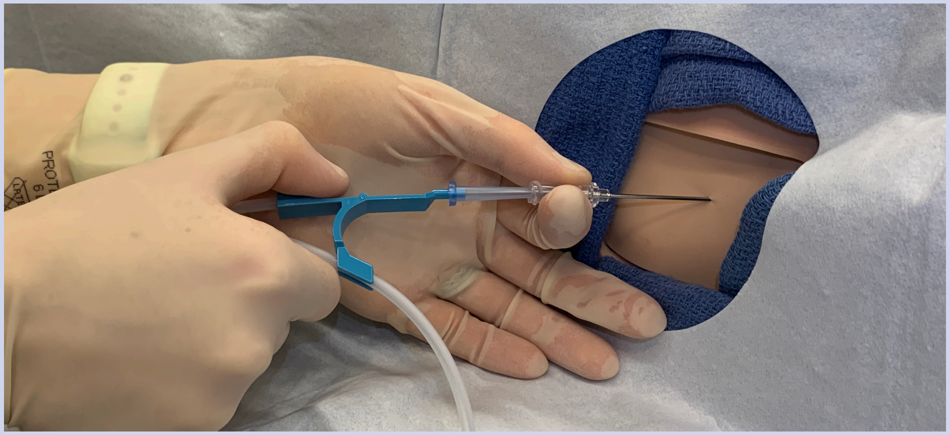

PIGTAIL CHEST TUBE – VUMC KIT
TECHNIQUE

Step 3: Remove needle

Hold the wire in place

Remove the finder needle from the chest

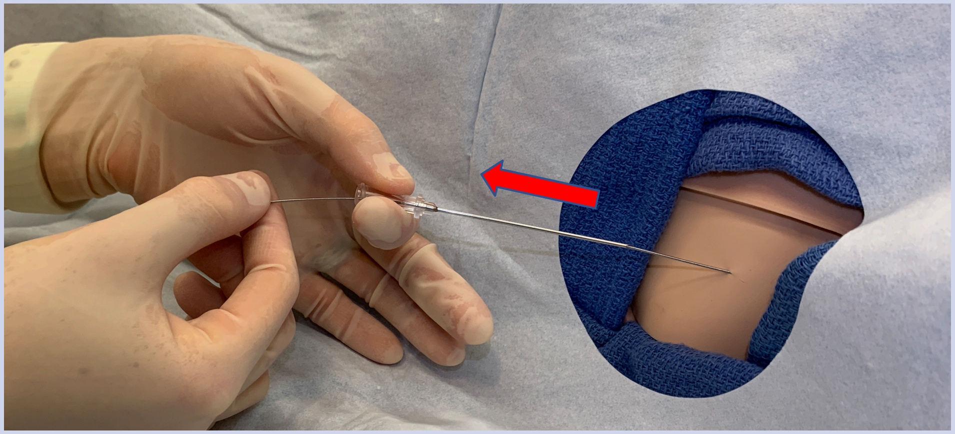

Step 4: Skin incision with scalpel

Hold the wire in place

Perform a small skin incision with the scalpel, approximately the width of the chest tube

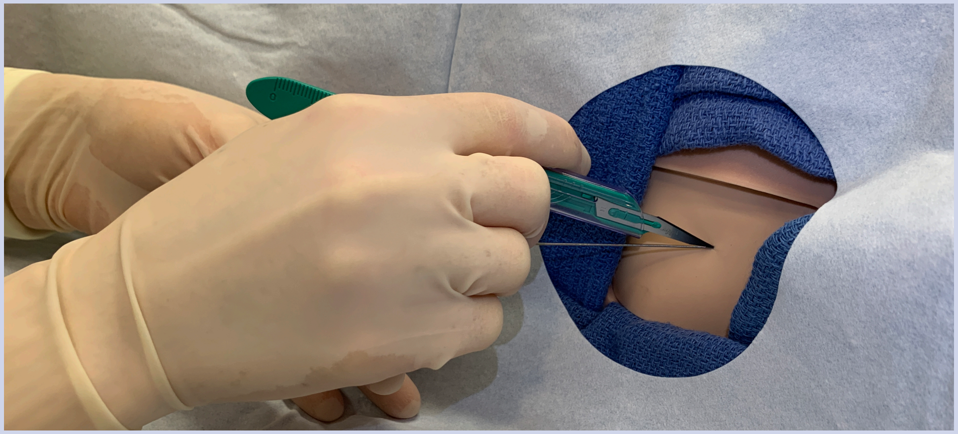

PIGTAIL CHEST TUBE – VUMC KIT
TECHNIQUE

Step 5: Insert the larger dilator

Thread the larger dilator over the wire

Insert in a slow and steady fashion, and twist the catheter as it enters the chest

**This torque will allow for creation of a tract through the subcutaneous tissues that will allow for easier passage of your catheter*

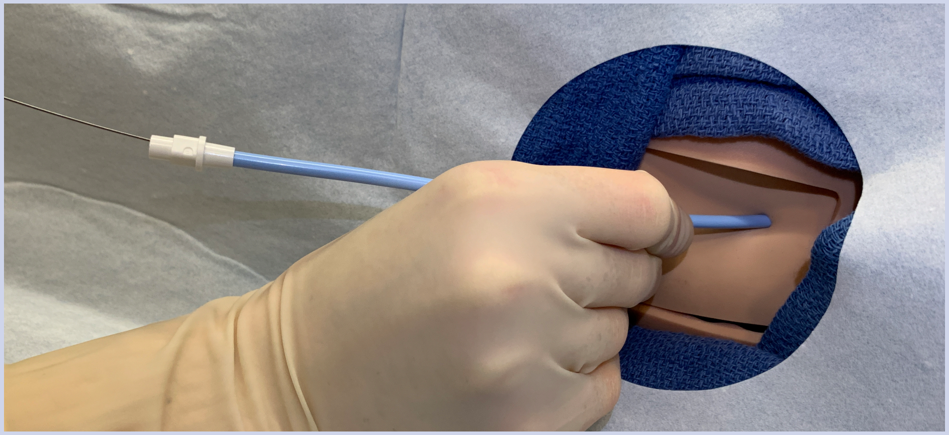

Step 6: Insert the small dilator into the trocar

Insert the smaller thin dilator into the rigid metal trocar

* *This allows the tip of the rigid metal trocar to have a soft flexible tip* (☆)

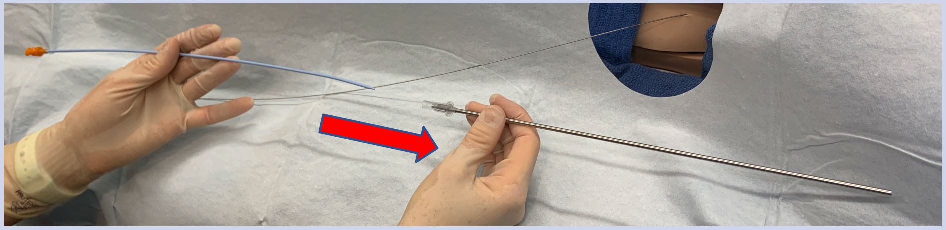

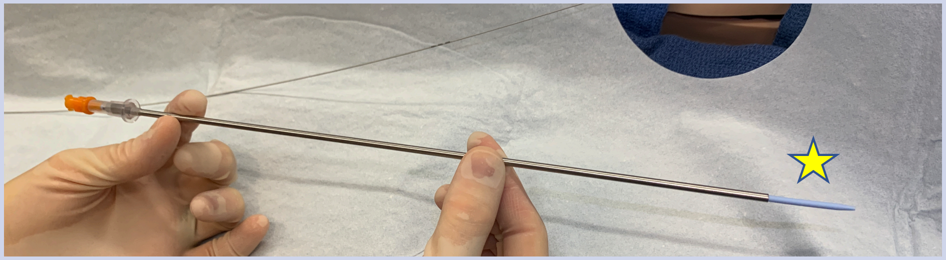

PIGTAIL CHEST TUBE – VUMC KIT
TECHNIQUE

Step 7: Insert the trocar into the chest tube

Insert the trocar/dilator combo into the chest tube

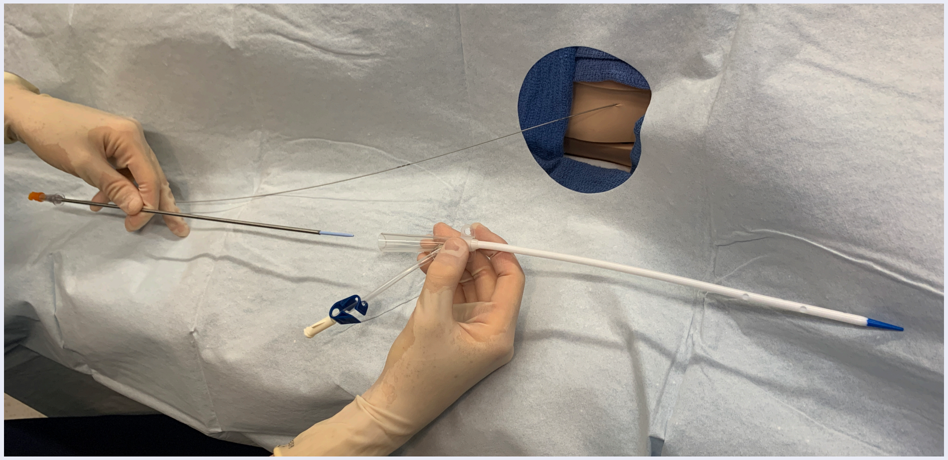

**Note the fenestrations at the insertion end of the chest tube → these must be placed inside the pleural space*

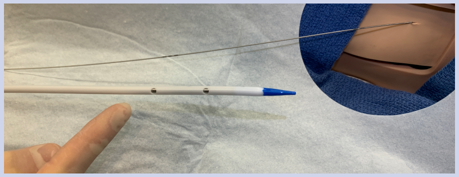

Step 8: Insert the chest tube + trocar/dilator combo

Thread the chest tube + trocar/dilator combo over the wire

Insert in a slow and steady fashion right at the chest wall to prevent kinking of the wire and allow for easy passage of the chest tube

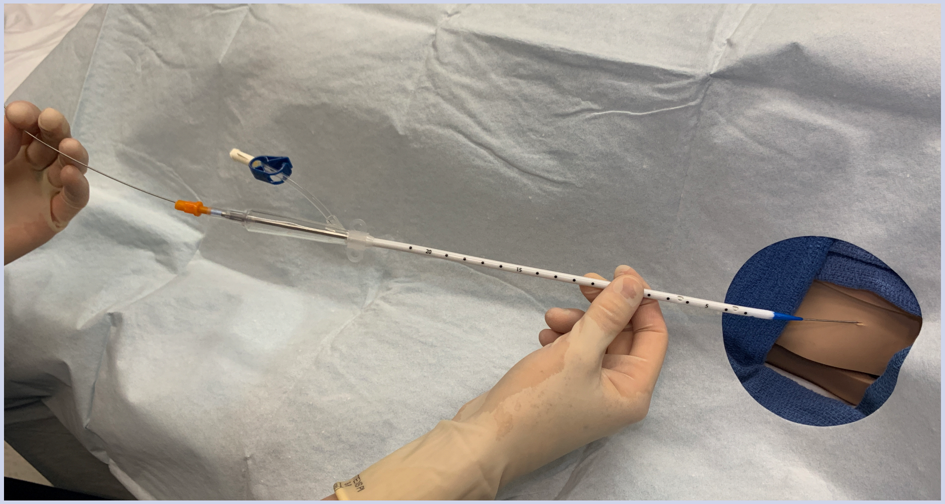

PIGTAIL CHEST TUBE – VUMC KIT
TECHNIQUE

Step 9: Stop the trocar → insert the chest tube

Once the tip of the trocar/dilator is in the pleural space, stop advancing the trocar/dilator

Keep the trocar/dilator where it is, and advance the chest tube forward into the pleural space

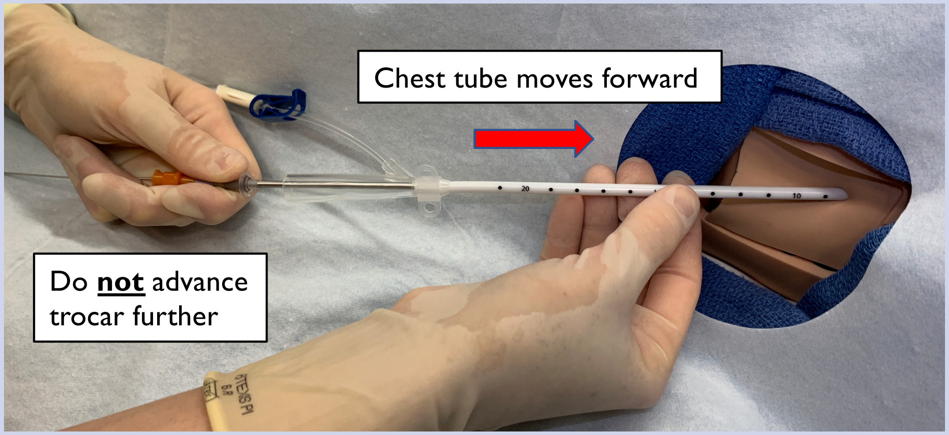

Step 10: Remove the trocar/dilator combo

Keep the chest tube in the pleural space, and remove the wire + trocar/dilator

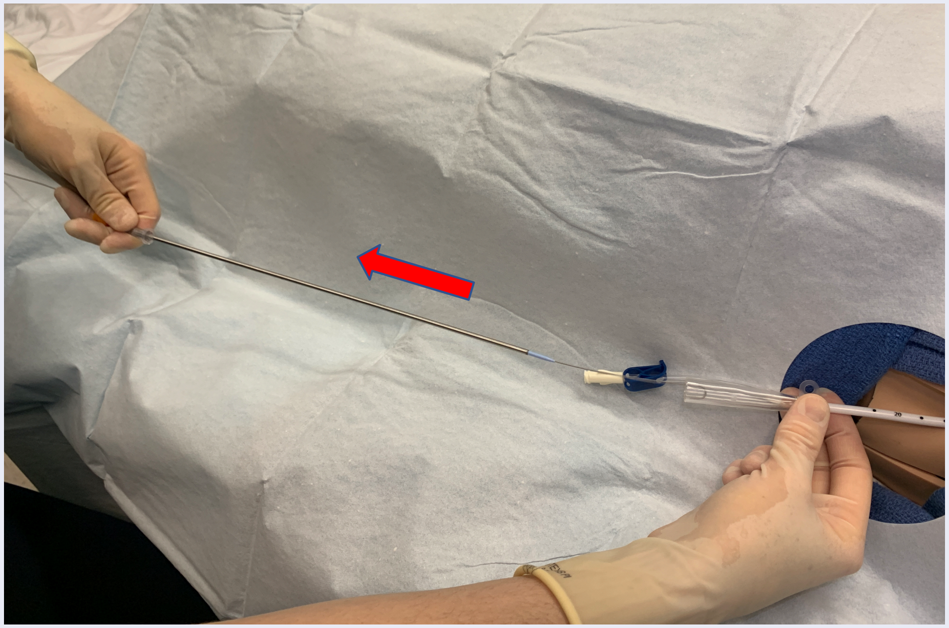

PIGTAIL CHEST TUBE – VUMC KIT
TECHNIQUE

Step 11: Attach the atrium or the blue cap

Option #1: Attach the atrium box for drainage	Option #2: Attach the blue cap (in the kit)
Place on the least amount of suction needed to re-expand the lung<br>• Ideally, initially place only on water seal (i.e. no suction), as there is no evidence to support routine initial use if suction for spontaneous PTX<br>• If incomplete resolution of the PTX → initiate @ -10 cm water suction<br>• If placed for fluid drainage → initiate @ -20 cm water suction	The blue cap is found in the kit and prevents drainage.
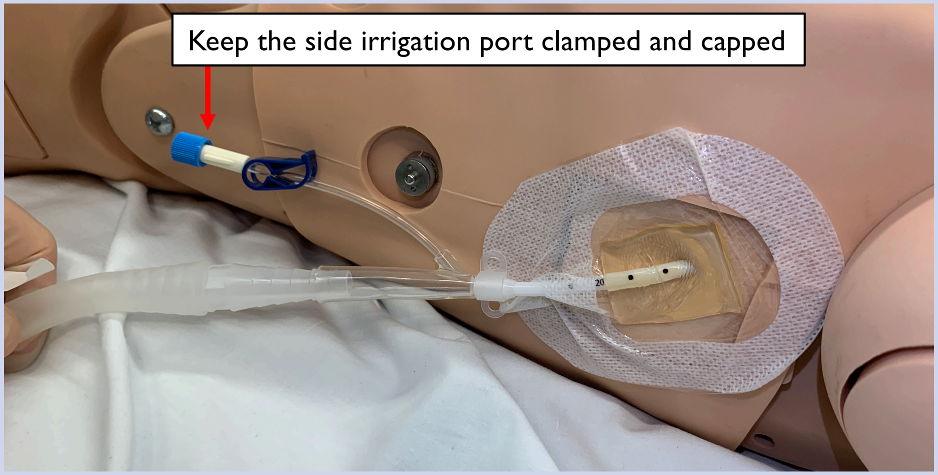 	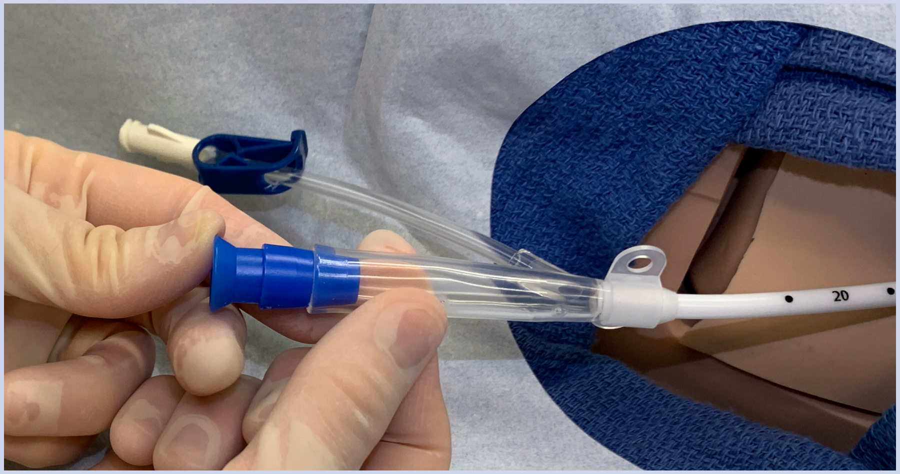

PIGTAIL CHEST TUBE – VUMC KIT
AFTERCARE

Step 12: Suture in place

Suture the chest tube in place through the anchor holes

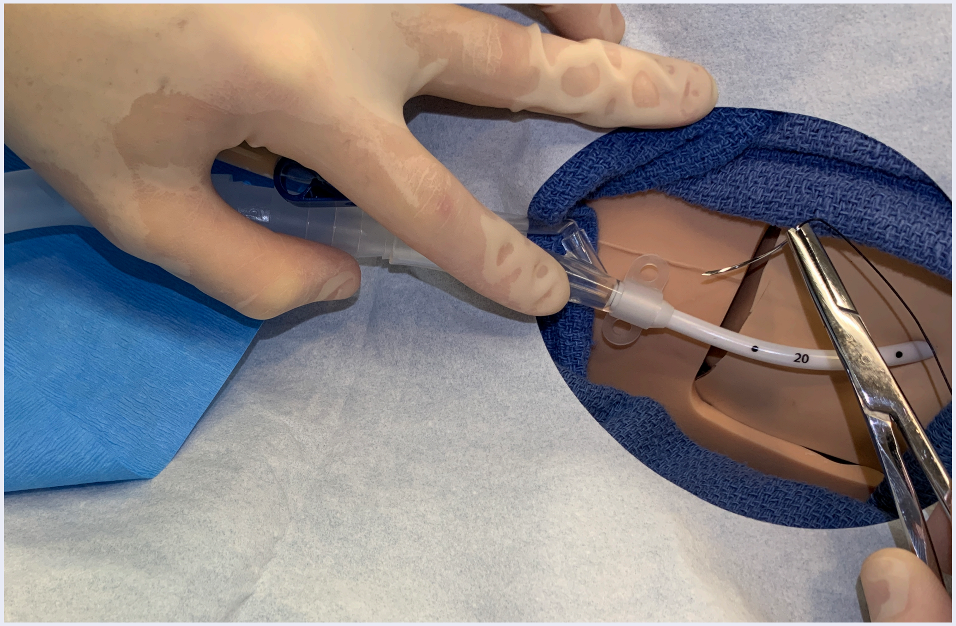

Step 13: Place the dressing

1) Place the Tegaderm around the chest tube entry site

2) <u>Optional:</u> you may also use silk tape over the atrium tubing to create a "mesentery" (reduces tension on the chest tube)

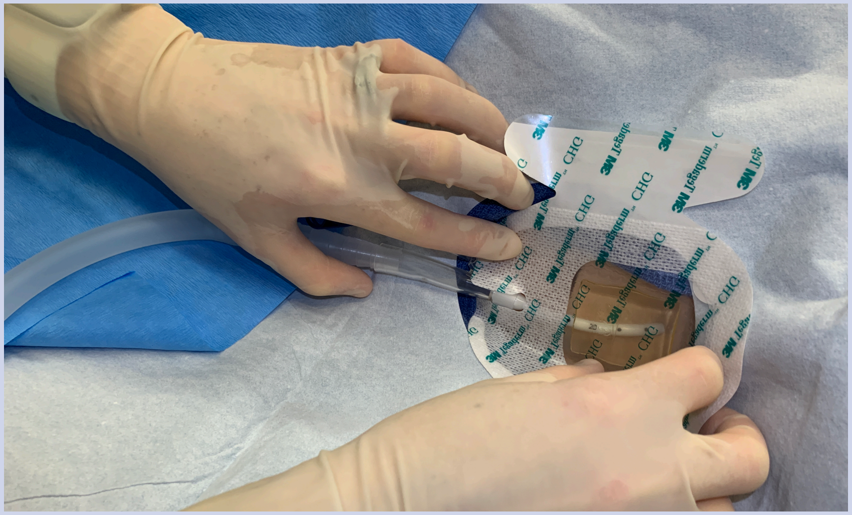

PIGTAIL CHEST TUBE – VUMC KIT
AFTERCARE

Don't forget to shoot a CXR!

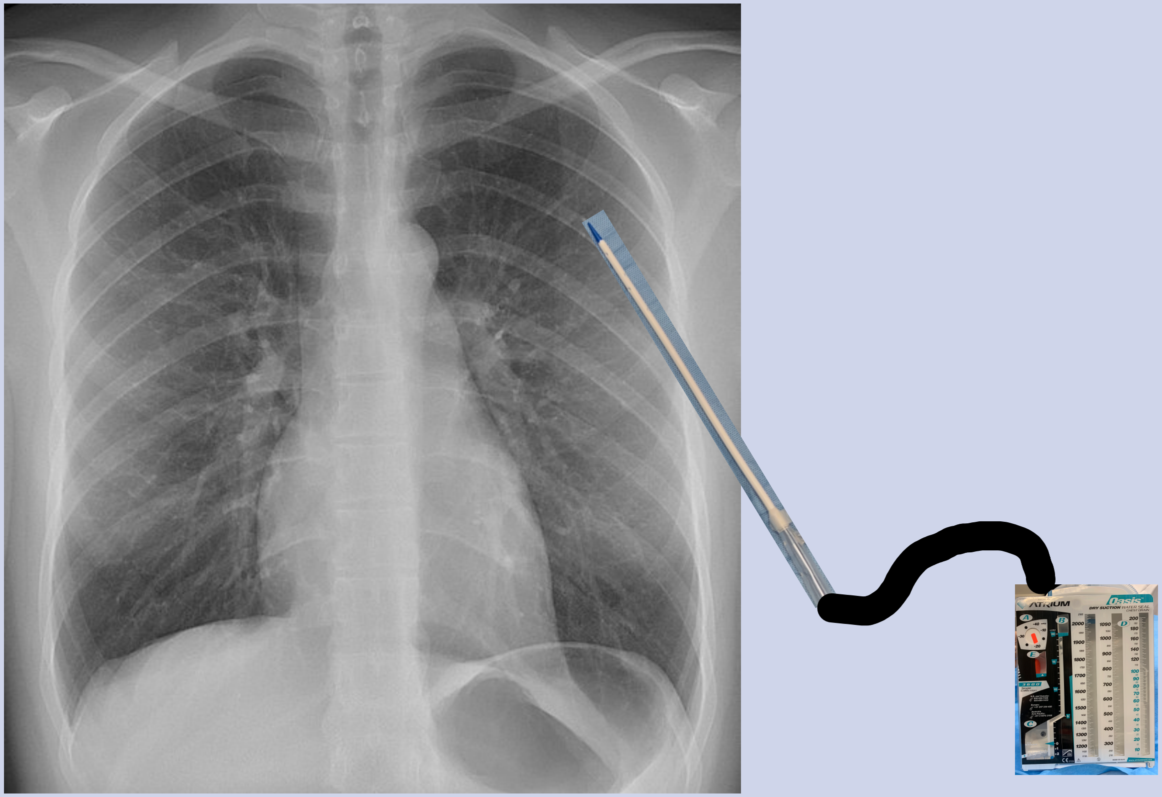

PIGTAIL CHEST TUBE – VUMC KIT
REFERENCES

1. MacDuff A, Arnold A, Harvey J. Management of spontaneous pneumothorax: British Thoracic Society Pleural Disease Guideline 2010. *Thorax* 2010; **65:**ii18-ii31
2. Tschopp JM, Bintcliffe O, Astoul P, et al. ERS task force statement: diagnosis and treatment of primary spontaneous pneumothorax. *Eur Respir J*. 2015 Aug;46(2):321-35.
3. Chang SH, Kang YN, Chiu HY, et al. A Systematic Review and Meta-Analysis Comparing Pigtail Catheter and Chest Tube as the Initial Treatment for Pneumothorax. *Chest*. 2018 May;153(5):1201-1212.
4. Ayed AK. Suction versus water seal after thoracoscopy for primary spontaneous pneumothorax: prospective randomized study. *Ann Thorac Surg*. 2003 May;75(5):1593-6.
5. Bauman ZM, Kulvatunyou N, Joseph B, et al. A Prospective Study of 7-Year Experience Using Percutaneous 14-French Pigtail Catheters for Traumatic Hemothorax/Hemopneumothorax at a Level-1 Trauma Center: Size Still Does Not Matter. *World J Surg*. 2018 Jan;42(1):107-113.
6. Wegerif G., Savage E.B. (2020) Chest Tube Thoracostomy. In: Rosenthal R., Rosales A., Lo Menzo E., Dip F. (eds) Mental Conditioning to Perform Common Operations in General Surgery Training. Springer, Cham. https://doi.org/10.1007/978-3-319-91164-9_8
7. "File:X-ray subtle pneumothorax in inspiration - annotated.jpg." Wikipedia Commons. https://commons.wikimedia.org/wiki/File:X-ray_subtle_pneumothorax_in_inspiration_-_annotated.jpg. This file is made available under the Creative Commons CC0 1.0 Universal Public Domain Dedication. https://creativecommons.org/publicdomain/zero/1.0/deed.en
8. "File:2313 The Lung Pleurea.jpg." Wikipedia Commons. https://commons.wikimedia.org/wiki/File:2313_The_Lung_Pleurea.jpg. This file is licensed under the Creative Commons Attribution 3.0 Unported license. https://creativecommons.org/licenses/by/3.0/deed.en. Adaptations made, annotated the anatomy and added an image of a syringe.
9. "File:201306 needle syringe.png." Wikipedia Commons. https://commons.wikimedia.org/wiki/File:201306_needle_syringe.png. This file is licensed under the Creative Commons Attribution 4.0 International license. https://creativecommons.org/licenses/by/4.0/deed.en. Adaptation made, pasted it on top of another image.
10. "File:Normal posteroanterior (PA) chest radiograph (X-ray).jpg." Wikipedia Commons. https://commons.wikimedia.org/wiki/File:Normal_posteroanterior_(PA)_chest_radiograph_(X-ray).jpg. This file is made available under the Creative Commons CC0 1.0 Universal Public Domain Dedication. https://creativecommons.org/publicdomain/zero/1.0/deed.en.
